# Agreement between muscle saturation breakpoints and lactate thresholds: Muscles comparison and sex difference in world‐class Nordic skiers

**DOI:** 10.1113/EP093311

**Published:** 2026-02-11

**Authors:** Jonas Forot, Forrest Schorderet, Cyril Burdet, Franck Brocherie, Grégoire P. Millet

**Affiliations:** ^1^ Institute of Sport Sciences University of Lausanne Lausanne Switzerland; ^2^ National Ski Nordic Center, Research and Performance Prémanon France; ^3^ French Ski Federation Annecy France; ^4^ Laboratory Sport, Expertise and Performance (EA 7370) French Institute of Sport Paris France

**Keywords:** lactate threshold, near‐infrared spectroscopy (NIRS), nordic skiing

## Abstract

Near‐infrared spectroscopy (NIRS) has emerged as a potential alternative method for determination of breakpoints equivalent to lactate thresholds. However, the optimal NIRS location remains unclear, particularly in Nordic skiing, which requires both upper‐ and lower‐limb contributions. This study aimed to evaluate the feasibility and accuracy of NIRS‐derived breakpoints determination (i.e., BP1 and BP2) compared to first (LT1) and second (LT2) lactate thresholds and to compare different muscle sites in male and female world‐class Nordic skiers. Fifty‐two world‐class Nordic skiers (29 males, 23 females) performed an incremental treadmill test on roller skis. NIRS sensors were located simultaneously on four muscles: vastus lateralis (VL), biceps femoris (BF), biceps brachii (BB), and triceps brachii (TB). Oxygen saturation ($S_{mO_{2}}$) was collected and analysed to detect BP1 and BP2 vs. LT1 and LT2. First, BP1 was too often undetectable or inaccurately detected, suggesting an unsuitable practical use. Second, BP2 was detected in VL (88.5%), BF (96.2%) and BB (86.5%) but not in TB (24.1%). Third, there was a very good accuracy (i.e., bias [95% CI] in heart rate between BP2 and LT2 in VL (−0.6 bpm [−8.9, 7.8]), BF (+1.3 bpm [−2.8, 4.2]) and BB (+1.0 bpm [−7.5, 9.5]). Finally, no significant differences were found between male and female athletes. NIRS appears as an effective non‐invasive method for detecting breakpoint equivalent to LT2 in both male and female world‐class Nordic skiers, especially if positioned on both BB and BF.

## Abstract (highlights)

- **What is the central question of this study?**
  Can near‐infrared spectroscopy (NIRS) muscle oxygen saturation breakpoints (BP1, BP2) validly replace lactate thresholds (LT1, LT2) in world‐class Nordic skiers? Which muscles provide the best signal (vastus lateralis, biceps femoris, biceps brachii, triceps brachii)? Are there are sex‐related differences?
- **What is the main finding and its importance?**
  BP1 was unreliable, but BP2 closely matched LT2 when measured on biceps femoris and biceps brachii (small heart rate bias, high agreement), while triceps brachii performed poorly. No meaningful sex differences were found, likely due to low adipose tissue thickness in elite athletes. Practically, NIRS offers a non‐invasive field alternative to estimate LT2 in Nordic skiing, with recommended sensor placement on biceps femoris and biceps brachii.

## 1 INTRODUCTION

Cross‐country skiing and biathlon have been extensively studied in scientific literature (Holmberg, 2015; Sandbakk & Holmberg, 2017). As in most endurance sports, training in Nordic skiing is characterized by either a pyramidal or a polarized training intensity distribution (Foster et al., 2022), which requires a significant volume of training (Osborne et al., 2024) and an accurate determination of the intensity domains and of their lower and upper boundaries (Keir et al., 2022). This precise management of intensities/loads aims to avoid overtraining or undertraining (Talsnes et al., 2022). In Nordic skiing, the determination of the lactate thresholds (i.e., LT1 and LT2 for the first and second lactate threshold, respectively) is commonly performed for elite athletes (Rundell, 1995; Talsnes et al., 2021). Recently, the advent of near‐infrared spectroscopy (NIRS) technology to measure skeletal muscle tissue oxy/deoxygenation offers a new avenue for threshold determination (Barstow, 2019; Millet & Brocherie, 2024; van der Zwaard et al., 2021). Muscle oxygen saturation ($S_{mO_{2}}$) has been proposed as a surrogate for ventilatory thresholds (VT1, VT2) (Lin et al., 2020; Reinpõld et al., 2024; Rodrigo‐Carranza et al., 2021) or lactate thresholds (LT1, LT2) (Farzam et al., 2018; Feldmann et al., 2022; Fleitas‐Paniagua et al., 2024; Raleigh et al., 2018; Salas‐Montoro et al., 2022) in various endurance sports, including cycling, running and rowing. However, these studies used different approaches to detect $S_{mO_{2}}$‐derived breakpoints (BP1 and BP2); that is, through double linear regression (van der Zwaard et al., 2016), visual identification of a prolonged $S_{mO_{2}}$ decrease (Salas‐Montoro et al., 2022), detection of a plateau in oxyhaemoglobin increase (Rodrigo‐Carranza et al., 2021), or segmented regression analysis (Feldmann et al., 2022). Compared to different physiological thresholds, these BPs provide moderate‐to‐strong correlations for VT1 (*r* = 0.58–0.63, *P* < 0.001) (van der Zwaard et al., 2016), LT2 (*r* = 0.955, *P* < 0.001), (Salas‐Montoro et al., 2022), VT2 (*r* = 0.69, *P* < 0.05) (Rodrigo‐Carranza et al., 2021), and both VT1 and VT2 (Feldmann et al., 2022).

To date, conflicting findings have been reported in the literature. For instance, some studies (Possamai et al., 2024; Sendra‐Perez, Sanchez‐Jimenez et al., 2023) have reported weak or no significant correlation between $S_{mO_{2}}$‐derived BPs and traditional physiological thresholds such as the maximal lactate steady state (MLSS) and critical power (CP), highlighting potential NIRS limitations to delineate intensity domains across all exercise modalities. In Nordic skiing, research on the validity and reliability of NIRS‐derived thresholds remains limited. The technical complexity of this sport, which involves both upper and lower limbs, makes the determination of intensity domains even more challenging. Studies in other endurance sports have shown that $S_{mO_{2}}$ responses differ significantly depending on the muscle monitored, due to differences in oxygen extraction capacity between proximal and distal muscles or between large and small muscles (Sendra‐Perez et al., 2025). In Nordic skiing, the choice of sensor placement (e.g., vastus lateralis (VL), rectus femoris, triceps brachii (TB), latissimus dorsi) is therefore critical. However, no consensus exists on the optimal muscle for $S_{mO_{2}}$ measurements in cross‐country skiing and biathlon.

Sex‐related physiological differences may also influence $S_{mO_{2}}$‐derived BP detection, adding complexity to NIRS‐based intensity domain assessment. Female athletes typically exhibit higher $S_{mO_{2}}$ values and lower muscle deoxygenation than their male counterparts, due to greater vasodilatory capacity and reduced oxygen extraction (Raberin et al., 2024; Sendra‐Pérez, Priego‐Quesada et al., 2023). Additionally, adipose tissue thickness is known as an important confounding factor for NIRS measurement (Barstow, 2019), and greater subcutaneous adipose tissue in females can attenuate NIRS signals, potentially affecting $S_{mO_{2}}$ readings and BP detection accuracy (Craig et al., 2017). This may alter their correlation with physiological thresholds and may further modulate sex‐specific $S_{mO_{2}}$ responses, particularly in sports with both upper and lower limb contribution to locomotion. Overall, their impact remains unexplored in cross‐country skiing and biathlon.

Therefore, the present study aimed to compare $S_{mO_{2}}$‐derived breakpoints (BP1 and BP2) with LT1 and LT2 in world‐class cross‐country skiers and biathletes. Due to the nature of these sports, we particularly aimed to determine the differences in the BP accuracy, when compared to LTs, in both upper‐ and lower‐limb muscles. Finally, we tested the hypothesis that BP accuracy would be reduced in female athletes, due to their higher adipose tissue thickness.

## 2 METHODS

### 2.1 Ethical approval

The present study was conducted in accordance with the standards set by the *Declaration of Helsinki* (latest revision). All participants were fully informed about the nature, purpose and potential risks of the study and provided their written informed consent prior to participation. All personal data were anonymized to ensure confidentiality. The study was approved by the local ethical committee (French National Conference of Research Ethics Committees; CPP Est I; approval number: 2014/33). The study was not registered in a public trials database, as it involved no intervention beyond routine physiological testing in elite athletes.

### 2.2 Participants

Fifty‐two (29 males and 23 females) athletes of the French national cross‐country skiing (*n* = 27) and biathlon (*n* = 25) teams were recruited for this study. According to the classification proposed by McKay et al. (2022), the sample represents 12 world‐class athletes, (Tier 5) such as Olympic or World Championship medalists, and 40 international‐level athletes, (Tier 4) such as national team members, typically within 7% of world‐leading performances.

All participants received detailed information about the study's objectives, procedures, risks and benefits and provided written informed consent prior to participation. Data were anonymized and processed in compliance with the General Data Protection Regulation (GDPR) to ensure confidentiality.

### 2.3 Procedure

Prior to testing, a 20‐min warm‐up was conducted at moderate intensity (i.e., below LT1) on a cycle ergometer (Wattbike, Wattbike Ltd, Nottingham, UK) to prepare the participants for the subsequent treadmill‐based roller skiing incremental test.

Briefly, the incremental test consisted in 3‐min work intervals interspersed by 15‐s rest intervals on a treadmill (Lode B.V., Groningen, The Netherlands) with continuous NIRS monitoring and physiological measurements (i.e., heart rate (HR) and lactate concentration ([La])), in order to determine BP1 and BP2, as well as LT1 and LT2, respectively, as well as maximal oxygen uptake (${\dot{V}}_{O_{2}\max}$) with a breath‐by‐breath metabolic cart (MetaMax 3B; Cortex Biophysik GmbH, Leipzig, Germany). The treadmill was inclined at a 7% slope, and the initial step started at 9.0 km/h with a further 1 km/h step increment. The test continued until voluntary exhaustion, with participants encouraged to sustain exercise as long as possible. During each rest interval, [La] was measured. Once LT2 was exceeded by one or two steps, the steps were shortened to 1‐min intervals without rest. Participants were instructed to ski with V2 technique roller skiing during the whole test.

### 2.4 Muscle oxygenation saturation

To measure $S_{mO_{2}}$, participants were equipped with four continuous‐wave NIRS devices (Moxy Monitor; Fortiori Designs LLC, Spicer, MN, USA). This device emits light at four wavelengths (630–850 nm) from a single LED, with two detectors (12.5 and 25 mm from the emitter) capturing scattered light (Feldmann et al., 2019; Fortiori‐Design, 2015). The penetration depth is approximately half the source–detector distance. A proprietary algorithm accounts for tissue light propagation and corrects Beer–Lambert law limitations, providing quantitative muscle oxygenation data (Barstow, 2019). Since light in micro‐vessels (>1 mm) is fully absorbed, reflected signals mainly originate from capillaries, reflecting oxygen supply and uptake (McCully & Hamaoka, 2000). The algorithm isolates muscle‐specific oxygenation, referred as $S_{mO_{2}}$, which is conceptually similar to tissue oxygen saturation or index (Barstow, 2019; Craig et al., 2017; Feldmann et al., 2022, 2019; Skotzke et al., 2024).

Sensors were placed on specific active skeletal muscles known for being important in cross‐country skating with the V2 technique, as identified in previous studies (Hesford et al., 2013; Holmberg, 2015, 2012; Stoggl & Born, 2021) and supported by a pilot study from our laboratory; that is, on the right VL (12 cm along the line from the anterior spina iliaca superior to the lateral side of the patella), on the right biceps femoris (BF, 17 cm along the line between the ischial tuberosity and the lateral epicondyle of the tibia), on the right biceps brachii (BB, at the most prominent bulge of the muscle) and on the right TB (at the most prominent bulge of the muscle) – all securely affixed and covered using medical adhesive tape to shield from potential ambient light intrusion.

### 2.5 Lactate thresholds

Capillary blood samples were drawn from a pinprick of the finger and analysed for [La] (Lactate Pro 2, Arkray, Japan). These samples were taken precisely at the end of each stage during the 15‐s rest period to ensure accurate measurement of lactate dynamics. Blood lactate levels were then analysed to determine LT1 and LT2 using the Dmax method (Cheng et al., 1992; Sendra‐Pérez, Encarnacion‐Martinez et al., 2023), which identified the point of maximal deviation from a straight line drawn between the first and last data points on the lactate‐intensity curve.

A Polar H10 HR monitor (Polar Electro, Kempele, Finland) was used to continuously recorded HR. Adipose tissue thickness was calculated with four skinfold thickness measured by caliper, as previously described (Durnin & Womersley, 1974).

### 2.6 Data analysis

${\dot{V}}_{O_{2}\max}$ was determined as the highest value of oxygen consumption recorded during the test, computed from a 15‐s rolling average.

**FIGURE 1:**
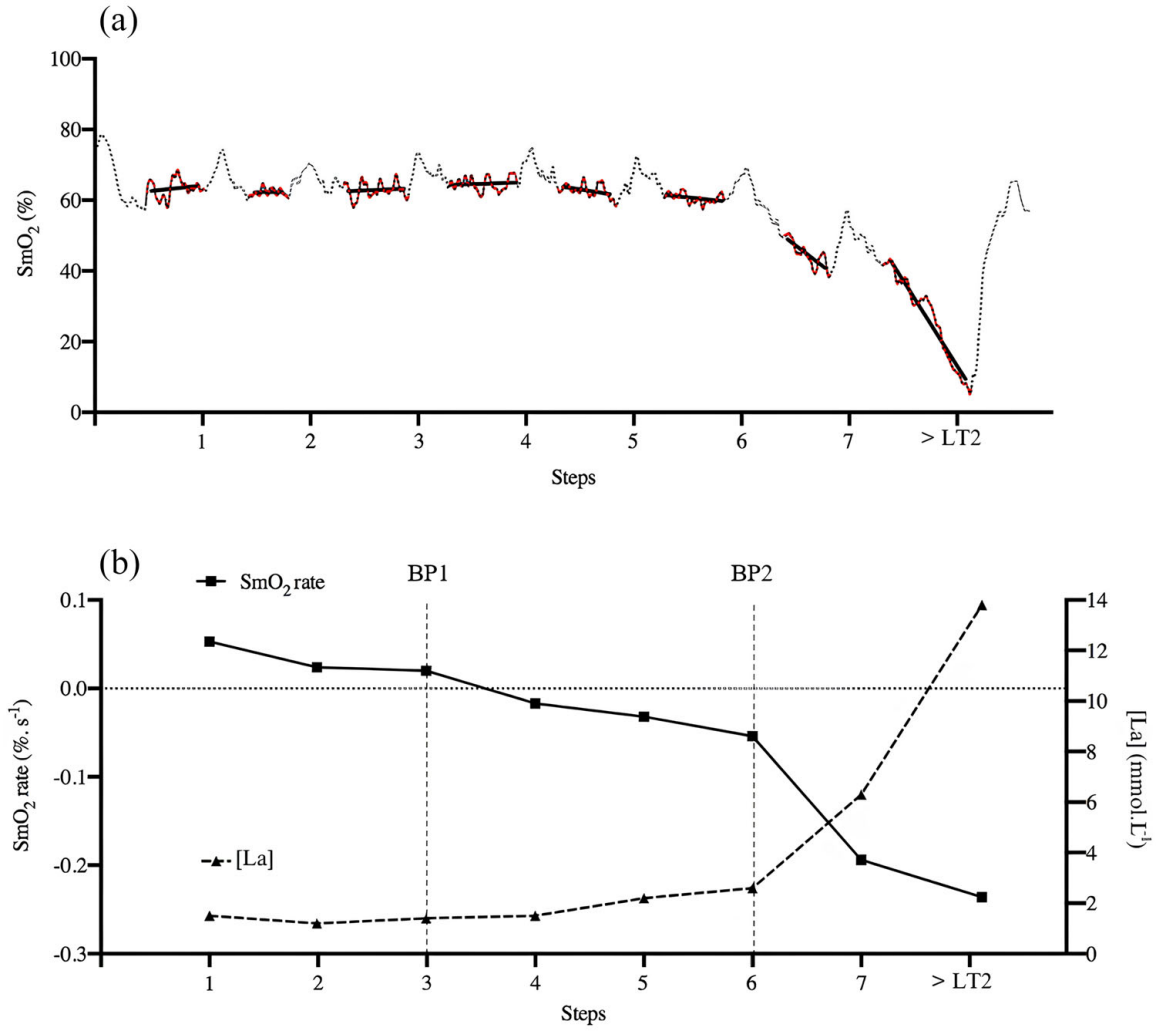
Representative NIRS figures. (a) Example data set from an incremental step test. Black dots indicate the momentary muscle oxygen saturation ($S_{mO_{2}}$) values, red dots represent the last 2 min of each step, and black lines illustrate the slope regression analysis for each step. (b) The relationship between blood lactate (mmol L^−1^) and test steps, along with the $S_{mO_{2}}$ rate (in % s^−^^1^) and steps. Breakpoints 1 and 2 are indicated by the dashed vertical lines.

BP were determined by analysing each work‐interval using multiple segmented linear regressions to evaluate the relationship between $S_{mO_{2}}$ and stage duration, expressed as $S_{mO_{2}}$ dynamics in %/min (Figure 1). Due to the 15‐s rest periods, only $S_{mO_{2}}$ data from the final 2 min of each 3‐min work‐interval were considered for the regression analysis. These $S_{mO_{2}}$ rate data were used for further analysis, as they more accurately reflect the stabilized oxygen dynamics during exercise, with the final 10 s of each work interval excluded to eliminate potential influences from early transitions into rest. If the maximal intensity step lasted less than 60 s, the previous segment was used as the final and maximal intensity step. $S_{mO_{2}}$ for each segment was plotted against stage duration to identify $S_{mO_{2}}$ rate BPs using a basic segmented regression analysis with two BPs, verified through visual inspection (Batterson et al., 2023).

The feasibility or visual control of using the method to determine the BPs indicated that, in certain cases, it was impossible to identify a BP. For BP1, there were sometimes no data plots showing a positive slope percentage, making impossible to determine BP1. For BP2, in some cases, there were no BPs in slopes, and the curves were very linear, making it impossible to determine BP2. In such cases, it was considered that determining the BP was not possible. These instances were accounted for in our statistics (Table 2) and included in the count of successful determinations for BP1 and BP2 for each participant and each muscle. But they were excluded from subsequent analyses when comparing BP1 and BP2 versus LT1 and LT2. Decisions were guided by visual inspection: in cases with a flat curve (zero slope), or more than two inflection points, no BP was retained. Only inflections showing clear and consistent changes in slope were accepted. No formal inter‐rater reliability assessment was conducted, although all curves were reviewed independently by two investigators, with disagreements resolved by consensus.

### 2.7 Statistical analysis

Females’ and males’ anthropometrical (i.e., age, height, body mass, body mass index, body fat, fat‐free body mass) and physiological characteristics (i.e., ${\dot{V}}_{O_{2}\max}$, LT1 and LT2 (%${\dot{V}}_{O_{2}\max}$), HR at LT1 and LT2) were compared using Student's paired *t*‐test.

To compare the possibility of determining BP1 or BP2, the chi‐square test of independence was used. The frequency of successful BP1 or BP2 (Yes/No) was compared between female and male participants. Pearson's 𝑟 correlations were calculated between the BP and LT differences (BP1 vs. LT1 and BP2 vs. LT2) in HR and %${\dot{V}}_{O_{2}\max}$ and body fat percentage or other anthropometric measures.

Repeated measures ANOVA was used to compare LT1 and BP1, as well as LT2 and BP2, across the different muscles. In cases where the assumptions of normality and homogeneity of variances were not met, Friedman's test was applied. *Post‐hoc* Bonferroni analyses were conducted to identify significant differences between pairs of methods.

The level of determination of agreement between BPs and LTs was defined using the method of Bland and Altman (Bland & Altman, 1986). The differences between the measurements performed with the methods were drawn in relation to the mean values, and 95% of the differences were expected to lie between the two limits of agreement (LOA) (95% confidence interval [95% CI]), which were the mean difference ±2 SD, expressed as bias ×/÷ random error. The validity of BP1 and BP2 compared to LT1 and LT2, respectively, was assessed using the intraclass correlation coefficient (ICC, two‐way random model, absolute agreement). The standard error of measurement (SEM) was calculated as:

$$\mathrm{SEM}\;=\;\mathrm{SD}\;\times \sqrt{1-\mathrm{ICC}}$$

where SD is the standard deviation of differences. Lower SEM indicates greater measurement stability. The statistical analyses were performed using SigmaPlot 11.0 software (SSI, San Jose, CA, USA).

## 3 RESULTS

The anthropometrical and physiological characteristics of the participants are displayed in Table 1. Males exhibited significantly higher body mass (*P* = 0.001), fat‐free mass (*P* = 0.001), and ${\dot{V}}_{O_{2}\max}$ (*P* = 0.001), while females had a greater percentage of body fat (*P* = 0.001). Minor but significant differences were also observed for LT1 and LT2 expressed as %${\dot{V}}_{O_{2}\max}$ (LT1 *P* = 0.046 LT2 *P* = 0.041).

**TABLE 1:** Anthropometrical and physiological characteristics of the participants.

Characteristic	Female (*n* = 22)	Male (*n* = 29)	*P*
Age (years)	24.0 ± 2.4	25.7 ± 4.0	0.643
Body height (cm)	165 ± 5	179 ± 5	0.001
Body mass (kg)	56.9 ± 6.0	74.2 ± 6.2	0.001
Body mass index (kg/m^2^)	20.8 ± 1.5	23.2 ± 1.7	0.001
Body fat (%)	16.8 ± 3.8	9.9 ± 2.9	0.001
Fat‐free body mass (kg)	47.3 ± 4.5	66.9 ± 5.6	0.001
${\dot{V}}_{O_{2}\max}$ (mL/min/kg)	65.6 ± 5.8	78.3 ± 4.7	0.001
${\dot{V}}_{O_{2}\max}$/lean body mass (L/min/kg_lbm_) in skiing	87.0 ± 5.7	78.9 ± 6.1	0.001
LT1 (%${\dot{V}}_{O_{2}\max}$)	62.9 ± 5.5	61.8 ± 4.9	0.046
HR at LT1 (bpm)	156 ± 10	156 ± 12	0.998
LT2 (%${\dot{V}}_{O_{2}\max}$)	79.6 ± 4.0	78.6 ± 4.5	0.041
HR at LT2 (bpm)	177 ± 8	177 ± 9	0.915

All values are presented as mean ± SD. HR: heart rate; LT1: first lactate threshold; LT2: second lactate threshold; ${\dot{V}}_{O_{2}\max}$: maximal oxygen uptake; Kglbm: kg lean body mass.

### 3.1 Breakpoint determination

The determination of BP1 was more frequent in females than in males for VL (87.0% vs. 58.6%, *P* = 0.014; Table 2) but not for the three other muscles. Similarly, the determination of BP2 was more frequent in females for VL (95.7% vs. 86.2%, *P* = 0.044), while no difference was observed for BF (96.6% vs. 100%, *P* = 0.730), BB (96.6% vs. 78.3%, *P* = 0.473) and TB (44.8% vs. 21.7%, *P* = 0.757). When pooling the data, the prevalence of $S_{mO_{2}}$‐derived BP determination appears to be higher for BP2 (88.5%) than for BP1 (69.2%). Furthermore, applying the method to at least one muscle shows a higher success rate (100% for BP2 and 96.1% for BP1).

**TABLE 2:** Prevalence (success percentage) of breakpoints determination by sex and muscles.

	Muscle				
	VL	BF	BB	TB	At least one^b^
BP1 detection feasibility (%)					
Pooled	69.2%	84.6%	69.2%	21.2%	**96.1%**
Female	87.0%	87.0%	65.2%	21.7%	**95.7%**
Male	58.6%	86.2%	75.9%	24.1%	**96.6%**
*P* ^a^	0.069	0.742	0.183	0.611	0.801
BP2 detection feasibility (%)					
Pooled	88.5%	96.2%	86.5%	32.7%	**100.0%**
Female	95.7%	100.0%	78.3%	21.7%	**100.0%**
Male	86.2%	96.6%	96.6%	44.8%	**100.0%**
*P* ^a^	0.090	0.421	0.137	0.094	0.998

^a^ *P*‐values are for difference between males and females. ^b^‘At least one’ indicates when at least one muscle allows breakpoints detection. BP1, breakpoint 1; BP2, breakpoint 2; BB, biceps brachii; BF, biceps femoris; TB, triceps brachii; VL, vastus lateralis.

The percentage of determination of BP1 or BP2 was not related to sex (*P* = 0.345), weight (*P* = 0.221), or body fat percentage (*P* = 0.189).

**FIGURE 2:**
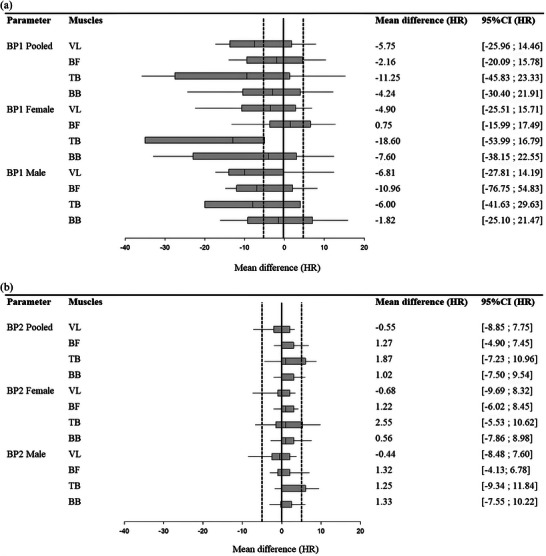
Box plot analysis showing mean differences (black squares) and 95% CI (thick darker horizontal lines) differences in HR at BP1 and LT1 (a) and BP2 and LT2 (b) for all athletes and for females and males. The vertical line inside the box indicates the median HR difference, representing the central value of the data. The lower and upper edges of the box represent the first quartile (Q1) (25th percentile) and the third quartile (Q3) (75th percentile) of the HR differences, respectively. The distance between Q1 and Q3 is the interquartile range (IQR). The lines extending from the box indicate the range of HR differences within 1.5× the IQR above Q3 and below Q1. They provide a sense of the spread of the central 50% of the data.

Figure 2 shows the mean differences in HR at BP1 and LT1 (Figure 2a) as well as between BP2 and LT2 (Figure 2b) for all participants and for females and males, respectively.

### 3.2 Sex differences

Small sex differences in HR offset between BP1/BP2 and LT1/LT2 were observed. At BP1, the HR difference relative to LT1 was slightly lower in females than in males for VL (−4.9 ± 10.2 vs. −6.8 ± 10.3 bpm, respectively). For TB, this offset was more pronounced in females (−18.6 ± 15.6 bpm) compared to males (−6.0 ± 16.5 bpm). At BP2, the HR difference relative to LT2 was slightly higher for BF in both females (+1.2 ± 3.6 bpm) and males (+1.3 ± 2.7 bpm), while TB exhibited a larger difference in females (+2.6 ± 3.9 bpm) compared to males (+1.3 ± 5.1 bpm). For BB, the HR offset remained moderate in males (+1.3 ± 4.4 bpm) showing a slightly higher difference than in females (+0.6 ± 4.2 bpm).

No correlation was found between BP1 (*r* = 0.08, *P* = 0.52) or BP2 (*r *= −0.05, *P* = 0.67) accuracy (i.e., HR differences to LT1 and LT2, respectively) and body fat percentage.

**FIGURE 3:**
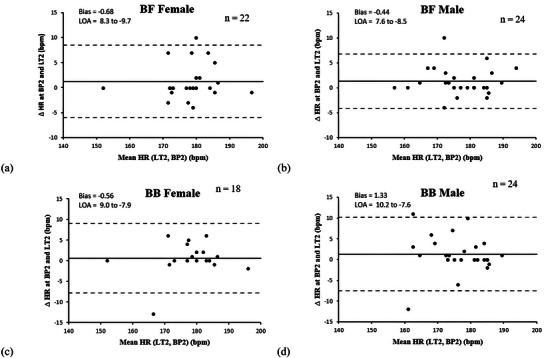
Bland–Altman plots analysis. Each plot shows the differences in heart rate between the second breakpoint (BP2) and the second lactic threshold (LT2) for biceps femoris (BF) or biceps brachii (BB) in females (a, c) or males (b, d), respectively. The black squares represent the bias, and the thick darker horizontal lines indicate the limits of agreement (LOA).

The Bland–Altman plots reveal small differences in HR biases between muscles and sexes (Figure 3), with a slight tendency for underestimation in females and slight overestimation in males, particularly for BB. The LOAs indicate small variability, suggesting that although the overall differences are small, there are notable individual variations.

It is noteworthy that only BF and BB muscles were selected for the Bland–Altman analysis because they showed the highest BP2 detection rates (96.2% and 86.5%, respectively) and the best accuracy compared to LT2. Additionally, these muscles represent both upper (BB) and lower (BF) limbs, which are crucial in cross‐country skiing and biathlon.

The reliability analysis of BP2 determination compared to LT2 across different muscles showed high ICC and low SEM, indicating strong agreement between the two methods. In males, the highest reliability was observed for BF (ICC = 0.954, SEM = 0.58 bpm), then for VL (ICC = 0.926, SEM = 1.09 bpm) and BB (ICC = 0.889, SEM = 1.48 bpm). Similarly, in females, ICCs remained high, with BF (ICC = 0.904, SEM = 1.11 bpm) showing the best agreement, followed by BB (ICC = 0.891, SEM = 1.37 bpm) and VL (ICC = 0.877, SEM = 1.57 bpm). The ICC for TB was not measured due to its low BP2 detection rate (32.7%), which limited the feasibility of reliable statistical analysis.

## 4 DISCUSSION

This study primarily aimed to evaluate (1) the possibility to determine BP1 and BP2 on four muscles; and (2) the accuracy of $S_{mO_{2}}$‐derived BPs when compared to LTs, in world‐class cross‐country skiers and biathletes. Results indicate that (1) the determination of the first $S_{mO_{2}}$‐derived breakpoint (BP1) is mostly unfeasible; (2) the second $S_{mO_{2}}$‐derived breakpoint (BP2) is more easily and accurately detectable when measured on BF and BB than on the other muscles; and (3) the accuracy of BP2, when compared to LT2, remains similar between females and males. Overall, these findings suggest that, despite limitations, NIRS can be used for the determination of BP2, equivalent to LT2 in world‐class Nordic skiers.

### 4.1 The determination of the first $S_{mO_{2}}$ breakpoint (BP1) is mostly unfeasible

The present results indicate the inability of using $S_{mO_{2}}$ to accurately determine BP1. Firstly, the success rate of BP1 detection varies across muscles. However, when considering at least one muscle, the overall success rate increases to 96.1%, mainly due to the higher detection rates in BF and BB. Secondly, there is a high inter‐individual variability in HR differences between BP1 and LT1, as shown by the large SDs displayed (−2.5 ± 6.4 bpm, −4.6 ± 10.5% for VL; +0.2 ± 5.2 bpm, +0.3 ± 8.1% for BF; −4.7 ± 7.9 bpm, −8.4 ± 13.9% for BB; and −10.3 ± 9.0 bpm, −18.3 ± 17.0% for TB; Figure 2a), which further limits the practical usefulness of NIRS for BP1 determination. Our results align with previous studies (Batterson et al., 2023; Eserhaut et al., 2025; Feldmann et al., 2022; Reinpõld et al., 2024; van der Zwaard et al., 2016) that also highlight no correlation between BP1 and LT1. Conversely, another study reported a strong relationship between BP1 and LT1 (Grassi et al., 1999). These contrasting findings may be due to the diversity of methodologies in NIRS‐derived BP determination (Feldmann et al., 2022; Rodrigo‐Carranza et al., 2021; Salas‐Montoro et al., 2022; van der Zwaard et al., 2016). As we opted for segmented regression analysis with visual inspection (Batterson et al., 2023), the comparison with previous experiments is rather limited and highlights the need for further development of a consensual and robust approach for using NIRS as a surrogate of LT1 determination.

### 4.2 Second breakpoint (BP2) determination is more accurate when measured on biceps brachial and BF than on the other muscles

The present results reveal that BP2 appears as an accurate surrogate of LT2. Firstly, the determination of BP2 showed high detection rates (88.5% for VL, 96.2% for BF, and 86.5% for BB), and a detection feasibility reaching 100% in at least one muscle. Secondly, the accuracy of BP2, when compared to LT2, was satisfying, as illustrated in Figure 2b with differences in HR and %${\dot{V}}_{O_{2}\max}$, respectively, between BP2 and LT2 of −0.55 ± 4.2 bpm and −8.9 ± 7.8% for VL, +1.27 ± 3.1 bpm and −4.9 ± 7.5% for BF, +1.87 ± 4.5 bpm and −7.2 ± 10.9% for TB, and 1.02 ± 4.3 bpm and −7.5 ± 9.5% for BB. In contrast, TB had a much lower detection rate (32.7%) and the differences with LT2 was much larger, indicating that TB is a less reliable muscle for NIRS‐derived BPs in elite Nordic skiers, despite its key role in cross‐country skiing (Stoggl & Born, 2021). This may arise by its high proportion of type‐II fibres (Holmberg, 2015; Johnson et al., 1973), which favours short and powerful contractions rather than sustained oxidative metabolism, as well as methodological challenges related to NIRS signal stability, arm movements and muscle compression.

As previously recommended (Arnold et al., 2024; Sendra‐Perez, Sanchez‐Jimenez et al., 2023; Stoggl & Born, 2021), we aimed to select at least one upper‐ and one lower‐limb muscle for further analysis. Results highlight BF and BB due to their higher reliability as illustrated in Figure 3. Although VL also appears suitable, particularly in females, selecting the same muscles (BF and BB) in both sexes facilitates the standardization of testing protocols for females and males for intensity monitoring and for sex‐difference analysis. The primary focus on VL reported in previous studies may relate to the sport‐specific context, mainly based on cycling protocols (Azevedo et al., 2022; Reinpõld & Rannama, 2023; Venckunas et al., 2024) that do not justify choosing BF (compression on the saddle) or upper‐limb muscles (low contribution).

Interestingly, we initially expected BP2 accuracy to be higher in agonist muscles (i.e., VL and TB), as they are directly involved in generating movement and experience higher metabolic demand in Nordic skiing (Holmberg, 2015; Stoggl & Born, 2021). However, our findings indicate that BP2 is actually more precise in antagonist muscles (i.e., BF and BB). This unexpected result underscores the importance of carefully considering muscle selection when using NIRS for physiological assessments, as factors such as movement artifacts (Siddiquee et al., 2018), compression effects and signal stability (Cooper et al., 2012) may influence measurement accuracy more than the metabolic demand per se. Additionally, differences in blood flow dynamics between muscles likely impact the NIRS signal quality (Bartlett et al., 2021), along with the presence of a thin adipose tissue layer over BF and BB, which may facilitate better light penetration and improve measurement reliability (Kirby et al., 2021; Vásquez Bonilla et al., 2023). Furthermore, in the lower limbs, the mono‐articular function of VL versus the biarticular role of BF could contribute to distinct oxygenation patterns, further justifying the choice of BF for more accurate $S_{mO_{2}}$‐derived BP detection. Although the VL is classically selected in NIRS studies due to its primary locomotor role, our results indicate that BF and BB provided a more reliable BP2 signal during roller‐ski skating. This may be explained by lower motion artefacts, reduced muscle compression, thinner adipose tissue layers and more stable oxygenation kinetics in these muscles compared with VL during treadmill roller skiing.

Although BP2 showed strong agreement with LT2 in the present study, conflicting results exist in the literature regarding the validity of NIRS‐derived BPs for intensity domain determination. Previous studies (Sendra‐Perez, Sanchez‐Jimenez et al., 2023; Venckunas et al., 2024) have reported a weak or non‐significant correlation between $S_{mO_{2}}$‐derived BPs and established physiological thresholds such as MLSS and CP. For instance, the low LOA between deoxygenated haemoglobin BP, $S_{mO_{2}}$‐BP and MLSS/CP compromises the interchangeability of these thresholds (Possamai et al., 2024). These results suggest that while NIRS‐derived BPs may align well with LTs in some cases, their use as a universal surrogate for all endurance training intensity markers remains uncertain. Further research is needed to explore the robustness of NIRS‐derived BTs in different endurance sports and exercise protocols, possibly by combining NIRS with other physiological markers (e.g., [La], ventilation) to improve BP determination accuracy and applicability in real‐world settings.

### 4.3 Sex differences

In line with previous studies (Solleiro Pons et al., 2024; van der Zwaard et al., 2016), no significant sex differences or effects of adipose tissue thickness on $S_{mO_{2}}$ measurements were observed within our sample. Solleiro‐Pons et al. (2024) attribute the absence of significant differences in oxygen uptake or extraction kinetics between sexes during moderate or heavy exercise intensities to the minimal adipose tissue in well‐trained individuals, which contributes in reducing inter‐individual variability. The performance level of our participants (i.e., Tiers 4–5), who generally have lower levels of adipose tissue thickness compared to the broader population (Larsson & Henriksson‐Larsen, 2008; Sandbakk et al., 2016), further reinforces these finding, due to the minimized variability and attenuated effects generally observed with NIRS measurements. Although small differences were observed, such as slightly higher BP2 detection rates in VL for females and in BF for males, these variations remained minimal and did not alter the overall interpretation and practical applications of the present findings. This suggests that NIRS could be a reliable device for highly trained or elite populations, where sex‐related differences in $S_{mO_{2}}$ measurements appear negligible. Overall, and contradictory to our hypothesis, there were no significant differences between females and males in the possibility of BP2 determination using NIRS. In Nordic skiing, elite female athletes rely heavily on upper‐body work, where sex differences are typically more pronounced than in the lower body. This training‐induced specialization may partly explain why BP determination feasibility did not differ between sexes, despite physiological differences in LT expression. This interpretation is supported by previous work showing that sex differences in endurance performance among elite cross‐country skiers are strongly influenced by upper‐body contribution, particularly during poling (Sandbakk et al., 2014).

### 4.4 Strengths and limitations

One of the main strengths of the present study is the large number of female and male world‐class‐level athletes (Tiers 5 and 4) involved, including many world and/or Olympic champions (in biathlon) and medalists (in cross‐country skiing). This study provides relevant and practical data, potentially transferable for field application. However, caution is required due to the inherent limitations of the Moxy‐derived measurements, which appear less robust than other NIRS devices (Crum et al., 2017; Feldmann et al., 2019). The use of the Moxy monitor represents both a strength and a limitation. While its portability and ease of use make it attractive for field‐based applications, its single‐source continuous‐wave technology provides lower spatial resolution than multi‐distance NIRS devices such as PortaMon (Artinis Medical Systems B.V., Elst, Netherlands), which may reduce measurement precision, particularly during high‐intensity exercise (McManus et al., 2018), but its applications in a training context remain underexplored.

It is important to note that the present findings are specific to world‐class Nordic skiers and biathletes, whose long‐term training induces a high reliance on upper‐limb musculature. In recreational or non‐skiing populations, BB oxygenation responses during running may be substantially lower, potentially limiting the relevance of this muscle for NIRS‐derived threshold detection.

Further limitations include the use of 3‐min stages during the incremental tests to accommodate the specific needs of such world‐class‐level participants, which could have potentially altered the precision of BP detection compared to longer stages (Rodrigo‐Carranza et al., 2021; Salas‐Montoro et al., 2022; van der Zwaard et al., 2016). Finally, caution is also needed for the transfer of these results to other sports and to athletes of lower calibres.

### 4.5 Practical application

In situ (i.e., in ecological settings) NIRS‐derived BPs could be used as surrogates of LT2, but not LT1, offering to the coaches and support staff a non‐invasive, easy‐to‐use and cost‐effective method compared to [La] measurements or gas exchange analysis. Our findings recommend positioning the NIRS device on both upper‐ and lower‐limb muscles (BB and BF, respectively), but the choice of a single muscle remains plausible, depending on the exercise (e.g., BB for double‐poling) or individual preferences. Of note, a preliminary study assessing NIRS positioning on the calf, abdominal and back muscles demonstrated excessive noise and poor data quality, mainly caused by artifacts and inadequate skin contact, leading us to conduct our measurements on VL, BF, BB and TB. Precise data on these muscles are valuable for Nordic skiing, highlighting the need to optimize device positioning for reliable measurements. Finally, in biathlon, BF is generally preferred over VL for field applications in order to not interfere with the prone shooting position. It should be emphasized that these recommendations are specific to cross‐country skiing and biathlon, where both upper‐ and lower‐limb musculature play a key role in performance. For other sports, however, the most relevant muscles for NIRS positioning may differ according to the sport‐specific movement patterns and primary muscles involved.

### 4.6 Perspectives

In cycling and middle‐distance running, training load can be accurately monitored using power meters or global‐positioning systems (GPS) (Bouillod et al., 2017; Gloersen et al., 2018). However, in cross‐country skiing and biathlon, terrain variability rather complicates load monitoring, as power meters are limited to upper‐limb measurements (e.g., pole sensors), and GPS‐based resistance estimations are imprecise due to variations in snow conditions (Gloersen & Gilgien, 2021; Zhao et al., 2023). While HR and [La] remain standard physiological markers (Sjodin et al., 1982), both have limitations. For example, [La] has slow recovery kinetics, making it less effective for real‐time adjustments, and HR is influenced by hydration, temperature and fatigue, affecting its reliability. Newer technologies, such as GPS and portable metabolic gas analysis systems, provide additional insights but remain costly, uncomfortable and impractical for routine field use. Unlike laboratory‐based methods, portable NIRS devices could provide on‐field assessments, helping bridge the gap between scientific precision and practical application. By allowing coaches and athletes to make immediate, data‐driven adjustments, NIRS could improve training load monitoring, particularly in cross‐country skiing and biathlon, where individualized load management is crucial for performance optimization. However, despite its potential, the integration of NIRS into training routines remains uncertain, requiring further research to validate its effectiveness and reliability in field settings (Crum et al., 2017; Feldmann et al., 2019; Klusiewicz et al., 2021; McManus et al., 2018).

Future studies combining NIRS with surface electromyography (EMG) during Nordic skiing would help clarify the respective contributions of muscle activation, perfusion and peripheral oxygen extraction to the determination of muscle oxygenation breakpoints.

### 4.7 Conclusion

This study highlights the potential of muscle oxygen saturation measurements for estimating breakpoints as surrogates of physiological thresholds in world‐class cross‐country skiers and biathletes. While the first breakpoint determination was not possible, the second breakpoint appears as a reliable surrogate of the second lactate threshold, particularly in the BF and BB. No significant sex differences were observed in breakpoint determination feasibility and accuracy. These findings support the use of NIRS as a practical, non‐invasive alternative to traditional physiological thresholds in endurance athletes, especially in sports requiring both upper‐ and lower‐limb contribution.

## AUTHOR CONTRIBUTIONS

Jonas Forot, Franck Brocherie, and Grégoire P. Millet conceived and designed the study. Jonas Forot, Forrest Schorderet, and Cyril Burdet collected the data. Jonas Forot performed the data analysis with input from Franck Brocherie and Grégoire P. Millet, and Jonas Forot drafted the manuscript. All authors critically revised the manuscript for important intellectual content. All authors have read and approved the final version of this manuscript and agree to be accountable for all aspects of the work in ensuring that questions related to the accuracy or integrity of any part of the work are appropriately investigated and resolved. All persons designated as authors qualify for authorship, and all those who qualify for authorship are listed.

## CONFLICT OF INTEREST

None declared.

## FUNDING INFORMATION

None.

## Data Availability

The data supporting the findings of this study are not publicly available due to ethical and confidentiality constraints related to elite athlete performance data. Anonymized data are available from the corresponding author upon reasonable request.

## References

[eph70212-bib-0001] Arnold, J. Y. , Hannah, N. V. H. , Martijn, K. , & Michael, S. (2024). Muscle reoxygenation is slower after higher cycling intensity, and is faster and more reliable in locomotor than in accessory muscle sites. Frontiers in Physiology, 15, 1449384. 39206382 10.3389/fphys.2024.1449384 PMC11349675

[eph70212-bib-0002] Azevedo, R. A. , Forot, J. , Millet, G. Y. , & Murias, J. M. (2022). Comparing muscle V̇o(2) from near‐infrared spectroscopy desaturation rate to pulmonary V̇o(2) during cycling below, at, and above the maximal lactate steady state. Journal of Applied Physiology, 132(3), 641–652. 35112926 10.1152/japplphysiol.00754.2021

[eph70212-bib-0003] Barstow, T. J. (2019). Understanding near infrared spectroscopy and its application to skeletal muscle research. Journal of Applied Physiology, 126(5), 1360–1376. 30844336 10.1152/japplphysiol.00166.2018

[eph70212-bib-0004] Bartlett, M. F. , Akins, J. D. , Oneglia, A. P. , Brothers, R. M. , Wilkes, D. , & Nelson, M. D. (2021). Impact of cutaneous blood flow on NIR‐DCS measures of skeletal muscle blood flow index. Journal of Applied Physiology, 131(3), 914–926. 34264131 10.1152/japplphysiol.00337.2021 PMC8526347

[eph70212-bib-0005] Batterson, P. M. , Kirby, B. S. , Hasselmann, G. , & Feldmann, A. (2023). Muscle oxygen saturation rates coincide with lactate‐based exercise thresholds. European Journal of Applied Physiology, 123(10), 2249–2258. 37261552 10.1007/s00421-023-05238-9

[eph70212-bib-0006] Bland, J. M. , & Altman, D. G. (1986). Statistical methods for assessing agreement between two methods of clinical measurement. The Lancet, 327(8476), 307–310. 2868172

[eph70212-bib-0007] Bouillod, A. , Pinot, J. , Soto‐Romero, G. , Bertucci, W. , & Grappe, F. (2017). Validity, sensitivity, reproducibility, and robustness of the PowerTap, Stages, and Garmin Vector power meters in comparison with the SRM device. International Journal of Sports Physiology and Performance, 12(8), 1023–1030. 27967278 10.1123/ijspp.2016-0436

[eph70212-bib-0008] Cheng, B. , Kuipers, H. , Snyder, A. C. , Keizer, H. A. , Jeukendrup, A. , & Hesselink, M. (1992). A new approach for the determination of ventilatory and lactate thresholds. International Journal of Sports Medicine, 13(07), 518–522. 1459746 10.1055/s-2007-1021309

[eph70212-bib-0009] Cooper, R. J. , Selb, J. , Gagnon, L. , Phillip, D. , Schytz, H. W. , Iversen, H. K. , Ashina, M. , & Boas, D. A. (2012). A systematic comparison of motion artifact correction techniques for functional near‐infrared spectroscopy. Frontiers in neuroscience, 6, 147. 23087603 10.3389/fnins.2012.00147 PMC3468891

[eph70212-bib-0010] Craig, J. C. , Broxterman, R. M. , Wilcox, S. L. , Chen, C. , & Barstow, T. J. (2017). Effect of adipose tissue thickness, muscle site, and sex on near‐infrared spectroscopy derived total‐[hemoglobin + myoglobin]. Journal of Applied Physiology, 123(6), 1571–1578. 28935822 10.1152/japplphysiol.00207.2017

[eph70212-bib-0011] Crum, E. M. , O'Connor, W. J. , Van Loo, L. , Valckx, M. , & Stannard, S. R. (2017). Validity and reliability of the Moxy oxygen monitor during incremental cycling exercise. European Journal of Sport Science, 17(8), 1037–1043. 28557670 10.1080/17461391.2017.1330899

[eph70212-bib-0012] Durnin, J. V. , & Womersley, J. (1974). Body fat assessed from total body density and its estimation from skinfold thickness: Measurements on 481 men and women aged from 16 to 72 years. British Journal of Nutrition, 32(1), 77–97. 4843734 10.1079/bjn19740060

[eph70212-bib-0013] Eserhaut, D. A. , Provost, J. A. , Ackerman, K. E. , & Fry, A. C. (2025). Monitoring skeletal muscle oxygen saturation kinetics during graded exercise testing in NCAA division I female rowers. Frontiers in Physiology, 16, 1538465. 40034535 10.3389/fphys.2025.1538465 PMC11873099

[eph70212-bib-0014] Farzam, P. , Starkweather, Z. , & Franceschini, M. A. (2018). Validation of a novel wearable, wireless technology to estimate oxygen levels and lactate threshold power in the exercising muscle. Physiological Reports, 6(7), e13664. 29611324 10.14814/phy2.13664 PMC5880957

[eph70212-bib-0015] Feldmann, A. , Ammann, L. , Gachter, F. , Zibung, M. , & Erlacher, D. (2022). Muscle oxygen saturation breakpoints reflect ventilatory thresholds in both cycling and running. Journal of Human Kinetics, 83, 87–97. 36157967 10.2478/hukin-2022-0054 PMC9465744

[eph70212-bib-0016] Feldmann, A. , Schmitz, R. , & Erlacher, D. (2019). Near‐infrared spectroscopy‐derived muscle oxygen saturation on a 0% to 100% scale: Reliability and validity of the Moxy monitor. Biomedical Optics, 24(11), 115001. 31741352 10.1117/1.JBO.24.11.115001 PMC7003144

[eph70212-bib-0017] Fleitas‐Paniagua, P. R. , de Almeida Azevedo, R. , Trpcic, M. , Murias, J. M. , & Rogers, B. (2024). Combining near‐infrared spectroscopy and heart rate variability derived thresholds to estimate the critical intensity of exercise. Journal of Strength and Conditioning Research, 38(1), e16–e24. 37815285 10.1519/JSC.0000000000004597

[eph70212-bib-0018] Fortiori‐Design, L. (2015). Introduction to muscle oxygen monitoring with Moxy. http://cdn2.hubspot.net/hub/188620/file‐433442739‐pdf/docs/moxy‐ebook‐intro‐to‐muscleoxygen.pdf?t=1488816603832

[eph70212-bib-0019] Foster, C. , Casado, A. , Esteve‐Lanao, J. , Haugen, T. , & Seiler, S. (2022). Polarized training is optimal for endurance athletes. Medicine & Science in Sports & Exercise, 54(6), 1028–1031. 35136001 10.1249/MSS.0000000000002871

[eph70212-bib-0020] Gloersen, O. , & Gilgien, M. (2021). Classification of cross‐country ski skating sub‐technique can be automated using carrier‐phase differential GNSS measurements of the head's position. Sensors (Basel), 21(8), 2705. 33921408 10.3390/s21082705 PMC8069750

[eph70212-bib-0021] Gloersen, O. , Kocbach, J. , & Gilgien, M. (2018). Tracking performance in endurance racing sports: Evaluation of the accuracy offered by three commercial GNSS receivers aimed at the sports market. Frontiers in Physiology, 9, 1425. 30356794 10.3389/fphys.2018.01425 PMC6189485

[eph70212-bib-0022] Grassi, B. , Quaresima, V. , Marconi, C. , Ferrari, M. , & Cerretelli, P. (1999). Blood lactate accumulation and muscle deoxygenation during incremental exercise. Journal of Applied Physiology, 87(1), 348–355. 10409594 10.1152/jappl.1999.87.1.348

[eph70212-bib-0023] Hesford, C. M. , Laing, S. , & Cooper, C. E. (2013). Using portable NIRS to compare arm and leg muscle oxygenation during roller skiing in biathletes: A case study. Advances in Experimental Medicine and Biology, 789, 179–184. 23852493 10.1007/978-1-4614-7411-1_25

[eph70212-bib-0024] Holmberg, H. C. (2015). The elite cross‐country skier provides unique insights into human exercise physiology. Scandinavian Journal of Medicine & Science in Sports, 25(S4), 100–109. 26589123 10.1111/sms.12601

[eph70212-bib-0025] Holmberg, L. J. (2012). Musculoskeletal Biomechanics in Cross‐country Skiing. [Dissertations, Linkoping University].

[eph70212-bib-0026] Johnson, M. A. , Polgar, J. , Weightman, D. , & Appleton, D. (1973). Data on the distribution of fibre types in thirty‐six human muscles. An autopsy study. Journal of the Neurological Sciences, 18(1), 111–129. 4120482 10.1016/0022-510x(73)90023-3

[eph70212-bib-0027] Keir, D. A. , Iannetta, D. , Mattioni Maturana, F. , Kowalchuk, J. M. , & Murias, J. M. (2022). Identification of non‐invasive exercise thresholds: Methods, strategies, and an online app. Sports Medicine, 52(2), 237–255. 34694596 10.1007/s40279-021-01581-z

[eph70212-bib-0028] Kirby, B. S. , Clark, D. A. , Bradley, E. M. , & Wilkins, B. W. (2021). The balance of muscle oxygen supply and demand reveals critical metabolic rate and predicts time to exhaustion. Journal of Applied Physiology, 130(6), 1915–1927. 33914662 10.1152/japplphysiol.00058.2021

[eph70212-bib-0029] Klusiewicz, A. , Rebis, K. , Ozimek, M. , & Czaplicki, A. (2021). The use of muscle near‐infrared spectroscopy (NIRS) to assess the aerobic training loads of world‐class rowers. Biology of Sport, 38(4), 713–719. 34937982 10.5114/biolsport.2021.103571 PMC8670802

[eph70212-bib-0030] Larsson, P. , & Henriksson‐Larsen, K. (2008). Body composition and performance in cross‐country skiing. International Journal of Sports Medicine, 29(12), 971–975. 18600606 10.1055/s-2008-1038735

[eph70212-bib-0031] Lin, C. W. , Huang, C. F. , Wang, J. S. , Fu, L. L. , & Mao, T. Y. (2020). Detection of ventilatory thresholds using near‐infrared spectroscopy with a polynomial regression model. Saudi Journal of Biological Sciences, 27(6), 1637–1642. 32489305 10.1016/j.sjbs.2020.03.005 PMC7254025

[eph70212-bib-0032] McCully, K. K. , & Hamaoka, T. (2000). Near‐infrared spectroscopy: What can it tell us about oxygen saturation in skeletal muscle?. Exercise and Sport Sciences Reviews, 28(3), 123–127. 10916704

[eph70212-bib-0033] McKay, A. K. A. , Stellingwerff, T. , Smith, E. S. , Martin, D. T. , Mujika, I. , Goosey‐Tolfrey, V. L. , Sheppard, J. , & Burke, L. M. (2022). Defining training and performance caliber: A participant classification framework. International Journal of Sports Physiology and Performance, 17(2), 317–331. 34965513 10.1123/ijspp.2021-0451

[eph70212-bib-0034] McManus, C. J. , Collison , J. , & Cooper , C. E. (2018). Performance comparison of the MOXY and PortaMon near‐infrared spectroscopy muscle oximeters at rest and during exercise. Journal of Biomedial Optics, 23(01), 1–14. 10.1117/1.JBO.23.1.015007 29368457

[eph70212-bib-0035] Millet, G. P. , & Brocherie, F. (2024). Permanent mechanical and physiological responses by biofeedback wearables: Worth the investment?. Journal of Applied Physiology, 137(3), 657. 39245280 10.1152/japplphysiol.00339.2024

[eph70212-bib-0036] Osborne, J. O. , Solli, G. S. , Engseth, T. P. , Welde, B. , Morseth, B. , Noordhof, D. A. , Sandbakk, O. , & Andersson, E. P. (2024). Annual volume and distribution of physical training in Norwegian female cross‐country skiers and biathletes: A comparison between sports, competition levels, and age categories‐The FENDURA project. International Journal of Sports Physiology and Performance, 19(1), 19–27. 37917966 10.1123/ijspp.2023-0067

[eph70212-bib-0037] Possamai, L. T. , Borszcz, F. K. , de Aguiar, R. A. , de Lucas, R. D. , & Turnes, T. (2024). Comparison of NIRS exercise intensity thresholds with maximal lactate steady state, critical power and rowing performance. Biology of Sport, 41(2), 123–130. 38524827 10.5114/biolsport.2024.129486 PMC10955745

[eph70212-bib-0038] Raberin, A. , Burtscher, J. , Citherlet, T. , Manferdelli, G. , Krumm, B. , Bourdillon, N. , Antero, J. , Rasica, L. , Malatesta, D. , Brocherie, F. , Burtscher, M. , & Millet, G. P. (2024). Women at altitude: Sex‐related physiological responses to exercise in hypoxia. Sports Medicine, 54(2), 271–287. 37902936 10.1007/s40279-023-01954-6 PMC10933174

[eph70212-bib-0039] Raleigh, C. , Donne, B. , & Fleming, N. (2018). Association between different non‐invasively derived thresholds with lactate threshold during graded incremental exercise. International Journal of Exercise Science, 11(4), 391–403. 29541332 10.70252/BUCT5185 PMC5841671

[eph70212-bib-0040] Reinpõld, K. , & Rannama, I. (2023). Oxygen uptake and bilaterally measured vastus lateralis muscle oxygen desaturation kinetics in well‐trained endurance cyclists. Journal of Functional Morphology and Kinesiology, 8(2), 64. 37218860 10.3390/jfmk8020064 PMC10204511

[eph70212-bib-0041] Reinpõld, K. , Rannama, I. , & Port, K. (2024). Agreement between ventilatory thresholds and bilaterally measured vastus lateralis muscle oxygen saturation breakpoints in trained cyclists: Effects of age and performance. Sports, 12(2), 40. 38393260 10.3390/sports12020040 PMC10892087

[eph70212-bib-0042] Rodrigo‐Carranza, V. , Gonzalez‐Mohino, F. , Turner, A. P. , Rodriguez‐Barbero, S. , & Gonzalez‐Rave, J. M. (2021). Using a portable near‐infrared spectroscopy device to estimate the second ventilatory threshold. International Journal of Sports Medicine, 42(10), 905–910. 33525000 10.1055/a-1343-2127

[eph70212-bib-0043] Rundell, K. W. (1995). Treadmill roller ski test predicts biathlon roller ski race results of elite U.S. biathlon women. Medicine & Science in Sports & Exercise, 27(12), 1677–1685. 8614325

[eph70212-bib-0044] Salas‐Montoro, J. A. , Mateo‐March, M. , Sanchez‐Munoz, C. , & Zabala, M. (2022). Determination of second lactate threshold using near‐infrared spectroscopy in elite cyclists. International Journal of Sports Medicine, 43(8), 721–728. 35021246 10.1055/a-1738-0252

[eph70212-bib-0045] Sandbakk, O. , Ettema, G. , & Holmberg, H. C. (2014). Gender differences in endurance performance by elite cross‐country skiers are influenced by the contribution from poling. Scandinavian Journal of Medicine & Science in Sports, 24(1), 28–33. 22621157 10.1111/j.1600-0838.2012.01482.x

[eph70212-bib-0046] Sandbakk, O. , Hegge, A. M. , Losnegard, T. , Skattebo, O. , Tonnessen, E. , & Holmberg, H. C. (2016). The physiological capacity of the world's highest ranked female cross‐country skiers. Medicine and Science in Sports and Exercise, 48(6), 1091–1100. 26741124 10.1249/MSS.0000000000000862 PMC5642331

[eph70212-bib-0047] Sandbakk, O. , & Holmberg, H. C. (2017). Physiological capacity and training routines of elite cross‐country skiers: Approaching the upper limits of human endurance. International Journal of Sports Physiology and Performance, 12(8), 1003–1011. 28095083 10.1123/ijspp.2016-0749

[eph70212-bib-0048] Sendra‐Pérez, C. , Encarnacion‐Martinez, A. , Oficial‐Casado, F. , Salvador‐Palmer, R. , & Priego‐Quesada, J. I. (2023). A comparative analysis of mathematical methods for detecting lactate thresholds using muscle oxygenation data during a graded cycling test. Physiological Measurement, 44(12), 125013. 10.1088/1361-6579/ad1457 38081136

[eph70212-bib-0049] Sendra‐Perez, C. , Encarnacion‐Martinez, A. , Salvador‐Palmer, R. , Murias, J. M. , & Priego‐Quesada, J. I. (2025). Profiles of muscle‐specific oxygenation responses and thresholds during graded cycling incremental test. European Journal of Applied Physiology, 125(1), 237–245. 39259396 10.1007/s00421-024-05593-1 PMC11752943

[eph70212-bib-0050] Sendra‐Perez, C. , Priego‐Quesada, J. I. , Salvador‐Palmer, R. , Murias, J. M. , & Encarnacion‐Martinez, A. (2023). Sex‐related differences in profiles of muscle oxygen saturation of different muscles in trained cyclists during graded cycling exercise. Journal of Applied Physiology, 135(5), 1092–1101. 37732376 10.1152/japplphysiol.00420.2023

[eph70212-bib-0051] Sendra‐Perez, C. , Sanchez‐Jimenez, J. L. , Marzano‐Felisatti, J. M. , Encarnacion‐Martinez, A. , Salvador‐Palmer, R. , & Priego‐Quesada, J. I. (2023). Reliability of threshold determination using portable muscle oxygenation monitors during exercise testing: A systematic review and meta‐analysis. Scientific Reports, 13(1), 12649. 37542055 10.1038/s41598-023-39651-z PMC10403529

[eph70212-bib-0052] Siddiquee, M. R. , Marquez, J. S. , Atri, R. , Ramon, R. , Perry Mayrand, R. , & Bai, O. (2018). Movement artefact removal from NIRS signal using multi‐channel IMU data. Biomedical Engineering Online [Electronic Resource], 17(1), 120. 30200984 10.1186/s12938-018-0554-9 PMC6131891

[eph70212-bib-0053] Sjodin, B. , Jacobs, I. , & Svedenhag, J. (1982). Changes in onset of blood lactate accumulation (OBLA) and muscle enzymes after training at OBLA. European Journal of Applied Physiology and Occupational Physiology, 49(1), 45–57. 6213407 10.1007/BF00428962

[eph70212-bib-0054] Skotzke, P. , Schwindling, S. , & Meyer, T. (2024). Side differences and reproducibility of the Moxy muscle oximeter during cycling in trained men. European Journal of Applied Physiology, 124(10), 3075–3083. 38809481 10.1007/s00421-024-05514-2 PMC11467065

[eph70212-bib-0055] Solleiro Pons, M. , Bernert, L. , Hume, E. , Hughes, L. , Williams, Z. J. , Burnley, M. , & Ansdell, P. (2024). No sex differences in oxygen uptake or extraction kinetics in the moderate or heavy exercise intensity domains. Journal of Applied Physiology, 136(3), 472–481. 38205552 10.1152/japplphysiol.00429.2023 PMC11213575

[eph70212-bib-0056] Stoggl, T. , & Born, D. P. (2021). Near infrared spectroscopy for muscle specific analysis of intensity and fatigue during cross‐country skiing competition‐A case report. Sensors, 21(7), 2535. 33916617 10.3390/s21072535 PMC8038464

[eph70212-bib-0057] Talsnes, R. K. , Moxnes, E. F. , Nystad, T. , & Sandbakk, O. (2022). The return from underperformance to sustainable world‐class level: A case study of a male cross‐country skier. Frontiers in Physiology, 13, 1089867. 36699686 10.3389/fphys.2022.1089867 PMC9870290

[eph70212-bib-0058] Talsnes, R. K. , Solli, G. S. , Kocbach, J. , Torvik, P. O. , & Sandbakk, O. (2021). Laboratory‐ and field‐based performance‐predictions in cross‐country skiing and roller‐skiing. PLoS ONE, 16(8), e0256662. 34428258 10.1371/journal.pone.0256662 PMC8384222

[eph70212-bib-0059] van der Zwaard, S. , Brocherie, F. , & Jaspers, R. T. (2021). Under the hood: Skeletal muscle determinants of endurance performance. Frontiers in Sports and Active Living, 3, 719434. 34423293 10.3389/fspor.2021.719434 PMC8371266

[eph70212-bib-0060] van der Zwaard, S. , Jaspers, R. T. , Blokland, I. J. , Achterberg, C. , Visser, J. M. , den Uil, A. R. , Hofmijster, M. J. , Levels, K. , Noordhof, D. A. , de Haan, A. , de Koning, J. J. , van der Laarse, W. J. , & de Ruiter, C. J. (2016). Oxygenation threshold derived from near‐infrared spectroscopy: reliability and its relationship with the first ventilatory threshold. PLoS ONE, 11(9), e0162914. 27631607 10.1371/journal.pone.0162914 PMC5025121

[eph70212-bib-0061] Vásquez Bonilla, A. A. , González‐Custodio, A. , Timón, R. , Camacho Cardenosa, A. , Camacho‐Cardenosa, M. , & Olcina, G. (2023). Training zones through muscle oxygen saturation during a graded exercise test in cyclists and triathletes. Biology of Sport, 40(2), 439–448. 37077776 10.5114/biolsport.2023.114288 PMC10108753

[eph70212-bib-0062] Venckunas, T. , Satas, A. , Brazaitis, M. , Eimantas, N. , Sipaviciene, S. , & Kamandulis, S. (2024). Near‐infrared spectroscopy provides a reproducible estimate of muscle aerobic capacity, but not whole‐body aerobic power. Sensors, 24(7), 2277. 38610488 10.3390/s24072277 PMC11014184

[eph70212-bib-0063] Zhao, S. , Lindinger, S. , Ohtonen, O. , & Linnamo, V. (2023). Contribution and effectiveness of ski and pole forces in selected roller skiing techniques on treadmill at moderate inclines. Frontiers in Sports and Active Living, 5, 948919. 36909359 10.3389/fspor.2023.948919 PMC9992420

